# A highly responsive NH_3_ sensor based on Pd-loaded ZnO nanoparticles prepared via a chemical precipitation approach

**DOI:** 10.1038/s41598-019-46247-z

**Published:** 2019-07-08

**Authors:** G. H. Mhlongo, D. E. Motaung, F. R. Cummings, H. C. Swart, S. S. Ray

**Affiliations:** 1 DST-CSIR National Centre for Nano-Structured Materials, Council for Scientific and Industrial Research, Pretoria, 0001 South Africa; 2 Department of Physics, University of the Free State, Bloemfontein, ZA9300 South Africa; 3 Electron Microscope Unit, University of the Western Cape, Bellville, 7535 South Africa; 4 Department of Applied Chemistry, University of Johannesburg, Doornfontein, 2028 Johanneburg South Africa

**Keywords:** Sensors and biosensors, Synthesis and processing

## Abstract

The gas-detecting ability of nanostructured ZnO has led to significant attention being paid to the development of a unique and effective approach to its synthesis. However, its poor sensitivity, cross-sensitivity to humidity, long response/recovery times and poor selectivity hinder its practical use in environmental and health monitoring. In this context, the addition of noble metals, as dopants or catalysts to modify the ZnO surface has been examined to enhance its sensing performance. Herein, we report preparation of Pd-loaded ZnO nanoparticles via a chemical precipitation approach. Various Pd loadings were employed to produce surface-modified ZnO nanostructure sensors, and their resulting NH_3_ sensing capabilities both in dry and humid environments were investigated. Through a comparative gas sensing study between the pure and Pd-loaded ZnO sensors upon exposure to NH_3_ at an optimal operating temperature of 350 °C, the Pd-loaded ZnO sensors were found to exhibit enhanced sensor responses and fast response/recovery times. The influence of Pd loading and its successful incorporation into ZnO nanostructure was examined by X-ray diffraction, high resolution-transmission electron microscopy, and X-ray photoelectron spectroscopy. XPS studies demonstrated that in all samples, Pd existed in two chemical states, namely Pd° and Pd^2+^. The possible sensing mechanism related to NH_3_ gas is also discussed in detail.

## Introduction

Due to the ongoing issue of elevated atmospheric pollution, the development of effective and inexpensive systems for the detection and quantification of environmentally hazardous gases is of particular importantance. Currently, standard air pollution measurements are still based on slow and costly analytical techniques, including gas chromatography and optical spectroscopy. Among other potential gas sensor technologies, chemi-resistive gas sensors have shown significant promise^1,2^. These sensors function through variations in their electrical resistance upon chemical reactions and/or adsorption between the target gas molecules and the semiconductor metal oxide (SMO). Indeed, they present a number of advantages over conventional bulky methods, including low cost, simple operation, and reliability as real-time control systems, all of which have led to their practical application in fields such as environmental monitoring, transportation, security, defence, space missions, agriculture, and health^1–3^. The first sensor device based on SnO_2_ was commercialised in 1968 as a sensor for domestic gas leaks^4^, and research into the use other SMOs, such as ZnO, TiO_2_, WO_3_, In_2_O_3_, etc., for the detection of environmentally hazardous gases has intensified since this discovery^1,2,5^. However, despite the research progress that has been made to demonstrate the advantages of SMOs as building blocks for gas sensors, a number of challenges remain in terms of attaining high sensitivity, low detection limits, fast response and recovery times, and a cross-sensitivity to humid atmospheres since an enhancement in sensitivity alone does not fulfil all of the ideal sensor requirements. Among the various SMOs examined to date, ZnO is the most frequently utilised n-type semiconductor material for gas sensing owing to its good chemical stability, which results in an excellent sensing capability for the detection of various pollutant/hazardous gases, such as hydrogen sulpfide, carbon monoxide, methane, and ammonia (NH_3_)^6–11^. Among these environmentally hazardous gases, NH_3_ is a highly toxic gas with a characteristic pungent smell. It is generally employed as a precursor for sewage treatment, foodstuffs, paper products, and fertilisers^12^. When inhaled over a prolonged period, it can cause serious respiratory diseases and can even lead to death. According to the Occupational Safety and Health Administratration (OSHA)^12^, NH_3_ has a permeable exposure limit of 50 ppm in the workplace. Due to its low density and corrosive nature, the leakage of NH_3_ can have serious consequences for the exposed areas. Thus, owing to the harmful effects of NH_3_ both on the environment and in the context of human health, urgent action must be taken to regularly control and monitor its trace levels in laboratories, factories, and public places.

As reported previously, the sensing properties of pristine nanostructured ZnO have been inhibited by poor sensitivity, cross-sensitivity to humidity, long response/recovery times, and poor selectivity^13^, thereby rendering it unsuitable for application as a reliable, robust, and accurate chemical gas sensor. Thus, to improve the sensing performances of ZnO-based sensing materials, a number of modifications have been examined, including the incorporation of dopants^14^, the dispersion of noble metal nanoparticles as sensitizers or catalysts on the ZnO surface^5–18^, the preparation of mixed SMOs nanocomposites^19^, crystal plane selection^20^, and surface defect control^14^. Although the introduction of metal additives has been validated as an effective approach in promoting the gas sensing capabilities of ZnO, the mechanism behind such enhanced sensing capabilities is not yet well understood. Similarly, Pd-loaded SMO nanostructures have not yet been extensively explored in the context of NH_3_ detection^5,21,22^. In addition, as relative humidity (RH) has been shown to influence the gas sensing properties of ZnO-based materials^13^, its influence should be considered when investigating the gas sensing properties of Pd-loaded ZnO based sensors.

In this context, we herein report a step-wise chemical precipitation approach to develop Pd-loaded ZnO NP-based sensors, with the ultimate aim of enhancing the sensor response performance in both dry and humid environments to allow their wider utilization. A comparison of the gas sensing properties of the pure and Pd-loaded ZnO NP-based sensors containing different quantities of Pd will also be conducted using NH_3_ as a probe and the possible sensing mechanism will be examined to account for the differences between the various materials. Finally, to further investigate the effect of Pd loading on the morphological, microstructural and optical prorperties of the ZnO NPs, various characterization techniques will be employed.

## Experimental Details

### Synthesis of pure ZnO nanoparticles (NPs)

**Figure 1:**
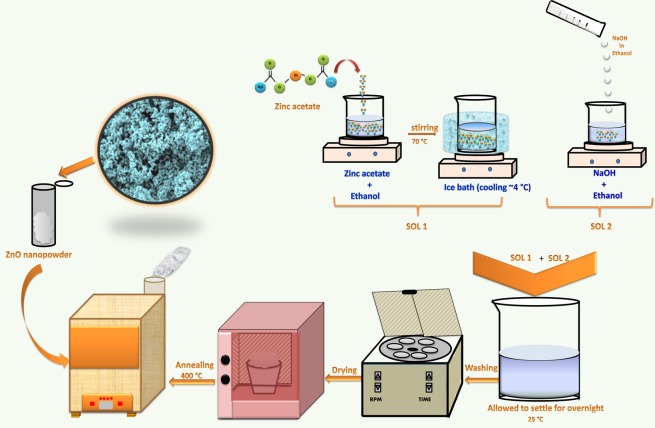
Schematic representation of the synthetic procedure employed for preparation of ZnO NPs.

All reagents employed were of analytical grade and were used as received from Sigma-Aldrich, South Africa. The pure ZnO NPs were synthesized via the following chemical precipitation approach: Zn(CH_3_COO)_2_ (0.5 g) was dissolved in 30 ml of boiling ethanol under stirring prior to cooling the resulting solution at 4 °C. In a separate beaker, a solution of NaOH (10 mmol) in ethanol was prepared, then added drop-wise to the Zn(CH_3_COO)_2_ solution with stirring to obtain a milky solution containing the ZnO NPs precipitate. Stirring was continued for 30 min and the suspension was maintained for 24 h. The resulting white precipitate was harvested by centrifugation, and repeatedly washed with heptane to remove any remaining impurities. After drying, the obtained white precipitate at 90 °C was annealed at 500 °C over 2 h to give pure ZnO NPs. The experimental procedure employed for preparation of the ZnO NPs is presented schematicaly in Fig. 1.

### Synthesis of the Pd-loaded ZnO NPs

To synthesise the Pd-loaded ZnO NPs, the above experimental procedure was followed, but with a few modifications. More specifically, the appropriate quantities of PdCl required to prepare 0.5, 0.75, and 1 mol% Pd-loaded ZnO NPs weres dissolved in ethanol, then added to hydrolysed Zn^2+^ precursor solution, prior to starring at room temperature for 24 h. The resulting Pd-loaded ZnO NPs precipitates were harvested by centrifugation and repeatedly washed with heptane to remove any remaining impurities. Finally, the obtained solid was dried at 90 °C, then annealed at 500 °C for 2 h.

### Sample characterisation

The synthesized pure ZnO and Pd-loaded ZnO NPs powders were characterised by X-ray diffractomery (XRD) (PANalytical X’pert PRO PW3040/60, PANalytical, the Netherlands) equipped with a Cu-Kα (λ = 1.5405 Ǻ) radiation source to examine their crystal phase and purity. Detailed structural and elemental distribution analyses were conducted using high resolution transmission electron microscope (HRTEM, FEI Tecnai G^2^20), operated at 200 kV, where the apparatus was equipped with a Fischione high angular annular dark-field detector (HAADF) and EDAX liquid nitrogen cooled Si(Li) detector, for scanning transmission electron microscopy (STEM) and energy dispersive X-ray spectroscopy (EDS) analyses, respectively. Drift corrected STEM coupled with EDS spectral images (STEM-EDS SI) were collected for the Pd-loaded ZnO NPs, using a 100 × 100 pixel frame size, with each pixel being 0.5 nm^2^ in size. Chemical state analyses of both the pure and Pd-loaded ZnO NPs were carried out by X-ray photoelectron spectroscopy (XPS) using a PHI 5000 Versaprobe-Scanning ESCA Microprobe with monochromatic Al-Kα radiation (*hν* = 1486.6 eV). The specific surface area studies of the NFs were measured using a Micrometirics TRISTAR 3000 surface area analyser. Preceding the analysis, the samples were degassed at 200 °C for 1 h under continuous flow of N_2_ gas to remove adsorbed impurities.

### Fabrication of the ZnO NPs-based sensors and their subsequent gas sensing tests

**Figure 2:**
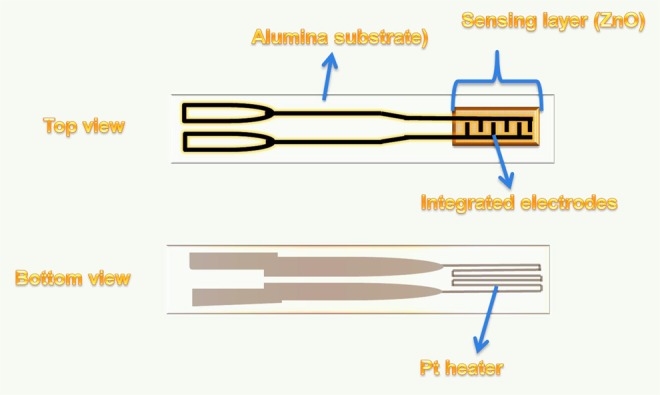
Schematic outline of the planar alumina substrate equipped with a Pt heater and Pt electrodes. The pure/Pd-loaded ZnO NP layers were deposited on top of the interdigitated electrodes. The heater maintained the sensor at the desired working temperature.

Fabrication process of the ZnO NPs-based sensors and their subsequent gas sensing tests were conducted according to our previously reported procedures^14^. More specifically, synthesized NPs (either pure or Pd-loaded ZnO NPs) were dispersed in ethanol to form a paste which were drop-coated onto an alumina substrates bearing a pair of platinum (Pt) electrodes on the top surface to provide electrical contacts, in addition to a micro-heater on the bottom surface (see Fig. 2). Prior to carrying out the gas testing measurements, the deposited NP layers were heated to 400 °C to achieve good adhesion. The gas sensing properties were then tested using a KSGAS6S gas sensing station (KENOSISTEC, Italy) under both dry and humid atmospheres at temperatures between 250 and 400 °C. The desired concentrations of NH_3_ gas (ppm) were established by variation of the flow rate ratios of synthetic air and NH_3_ gas. The electrical responses of the ZnO sensor devices were acquired by monitoring the electrical changes in resistance under a constant applied voltage during cyclic exposure to different NH_3_ gas concentrations using a Keithley 6487 picoammeter/voltage source meter.

## Results and Discussion

### Structural and morphological analysis

**Figure 3:**
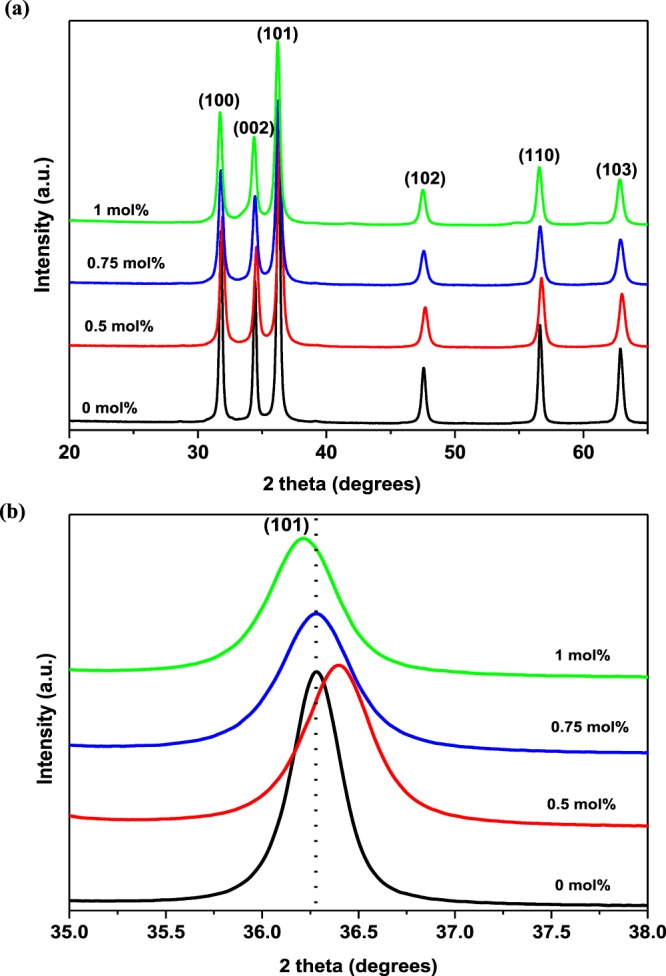
(**a**) XRD patterns of the pure and Pd-loaded ZnO NPs containing different Pd loadings. (**b**) Magnified XRD peaks corresponding to the (101) plane.

Following the successful synthesis of the pure and Pd-loaded ZnO NPs containing a range of Pd contents (i.e., 0.5, 0.75, and 1 mol%), their phase purities and crystal structures were examined using XRD. Figure 3(a) shows the diffraction patterns acquired for the pure and Pd-loaded ZnO samples, and these were readily indexed to the würtzite phase of ZnO, which corresponds to a hexagonal structure (JCPDS Card no. 36–1451). No additional diffraction peaks corresponding to Pd were observed; however, the intensities of the diffraction peaks decreased following Pd addition. This observation suggests deterioration of the ZnO lattice crystallinity upon the incorporation of Pd. Figure 3(b) illustrates a magnified diffraction peak corresponding to the (101) plane where an obvious shift to higher diffraction angles was observed with an initial Pd loading of 0.5 mol%. This was followed by a shift to low diffraction angles for higher Pd loadings (i.e., 0.75 and 1 mol%). Such shifting of the diffraction peak position is likely due to a range of parameters, including the inter-planar distance (*d*_*hkl*_), the average crystallite size (*D*), and the strain (ε) inside the lattice^14,23^. Considering that both the strain and the crystallite size are calculated as a function of the angular peak width at half maxima (*β*) and the diffraction peak position, the role of 2*θ* in determining these values is particularly small compared to that of β. The observed shift in diffraction peak positions towards higher and lower 2*θ* angles therefore indicates the shortening and elongation of the inter-planar distance, respectively, which in turn leads to compression and expansion of the ZnO lattice through the partial substitution of smaller ionic radii Zn^2+^ ions (0.074 nm) by larger ionic radii Pd^2+^ ions (0.080 nm), respectively^24^. In addition, the observed shifts correspond with previous reports of doped ZnO nanostructures, thereby confirming that these shifts strongly depend on the dopant concentration^14,25,26^. This behaviour also suggests that the introduction of Pd induces lattice strain in the ZnO lattice.

The average crystallite sizes of pure and Pd-loaded ZnO NPs were also determined by measuring the full width at half maximum (FWHM) of the intense (101) diffraction peak using the Debye-Scherrer formula^27^: The obtained crystallite sizes for the various samples are listed in Table 1, where it is apparent that the crystallite size decreases from 26 Å for the pure ZnO NPs to 20, 19, and 20 Å for Pd loadings of 0.5, 0.75, and 1 mol%. This can be attributed to distortion of the ZnO lattice by the larger Pd^2+^ ions, which constrains the growth and nucleation rate of the ZnO NPs.

**Table 1:** Crystallite size (D), strain (ε), and lattice constants (a and c) for the pure and Pd-loaded ZnO NPs containing different Pd loadings.

Sample	2ɵ	d_101_ (Å)	D (Å)	ε (10^−3^)	cell parameters (Å)	
(mol%)	(degrees)				a	c
0	36.28	2.473	26	4.22	3.2498	5.2062
0.5	36.43	2.464	20	5.66	3.2530	5.0806
0.75	36.27	2.474	19	5.97	3.2537	5.1596
1	36.21	2.478	20	5.67	3.2562	5.1931

As one of the key parameters contributing to the shift in diffraction peak position, the micro-strain was also calculated^25^. The increasing trend in the micro-strain values (see Table 1) with increasing Pd loading could be mainly attributed to the lattice distortion created by the dopant ions, as a result of lattice mismatch between the Pd^2+^ and Zn^2+^ ionic radii. Based on the observed results, the effect of Pd concentration on the crystallite structure was also investigated, and so the purpose lattice parameters were calculated for different Pd loadings. The lattice spacing values were calculated from the XRD data for the pure and Pd-loaded ZnO NPs (see Table 1) according to Bragg’s equation^28^. The lattice parameters calculated for the different Pd loadings are listed in Table 1, where it is apparent that the lattice parameter *a* increased upon increasing the dopant concentration, while lattice parameter *c* decreased under similar conditions. The increase in lattice parameter *a* with increasing Pd loading is likely due to the larger ionic radius of Pd^2+^ (0.80 Å) compared with that of Zn^2+^ (0.74 Å)^29^. Furthermore, the doping of foreign impurities into an SMO lattice bearing electrically active ions can induce changes in the lattice consants due to the effects of crystallite size^29^.

**Figure 4:**
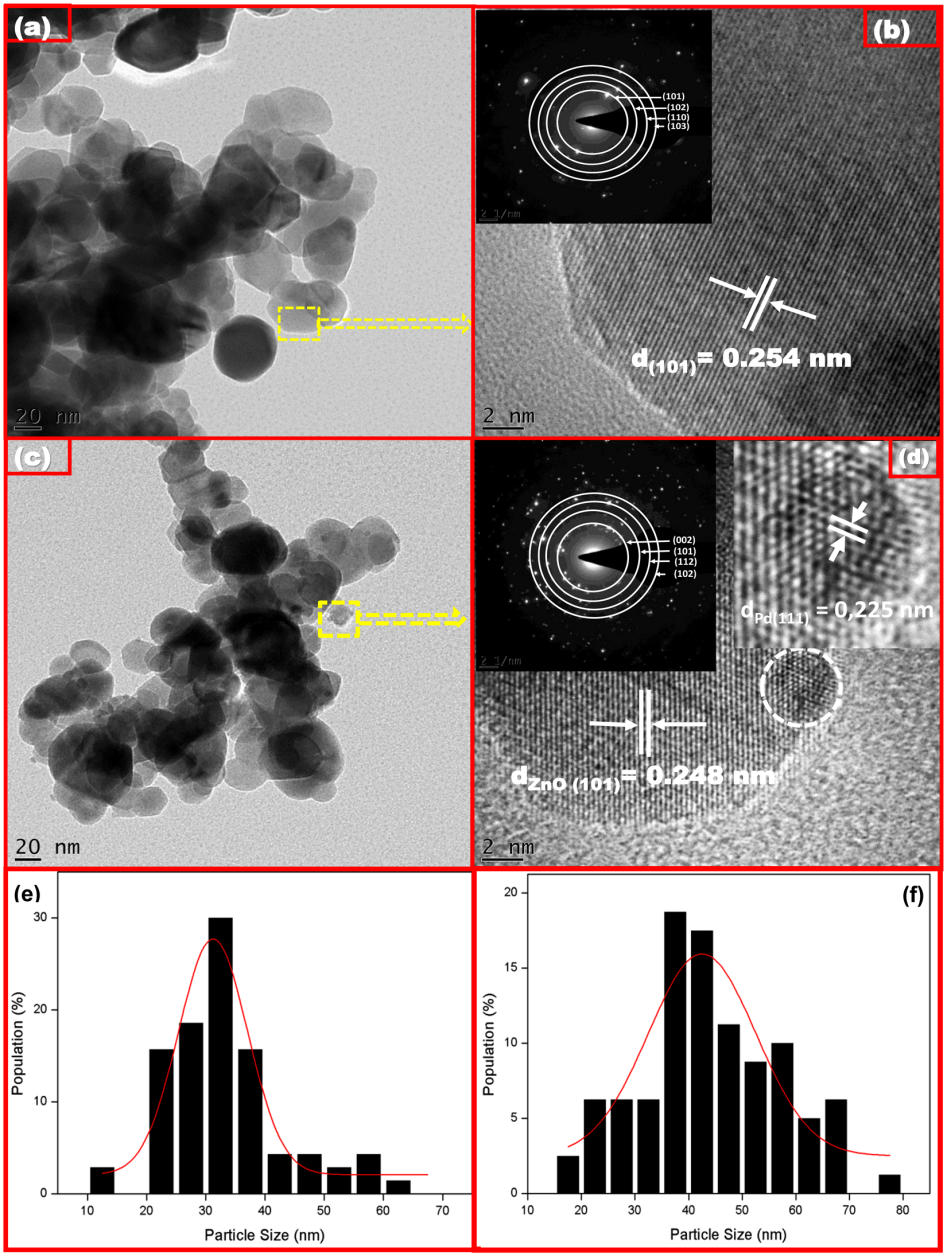
Bright-field TEM micrographs (**a**,**c**) and HRTEM micrographs (**b**,**d**) of pure ZnO and Pd-loaded (1 mol%) ZnO NPs. The insets in (**b**,**d**) respectively show the SAED patterns of the pure and Pd-loaded (1 mol%) ZnO NPs.

To examine the structural features of the Pd-loaded ZnO, TEM and HRTEM were employed. In this case, the ZnO sample containing 1 mol% Pd was selected for examination. As shown in Fig. 4(a,c), the TEM micrographs revealed particle-like features. However, it was also observed that following the addition of 1 mol% Pd, a random distribution of smaller-sized particles formed on the surfaces of the clustered ZnO particles. Moreover, the particle size distribution calculated from the TEM micrographs presented in Fig. 4(e,f) revealed a drecease in average particle size with Pd loading from 40 nm for pure ZnO to 31 nm for 1 mol% Pd-loaded ZnO. This observation corresponded with the XRD results where a decrease in crystallite size with Pd loading was observed.

Figure 4 Bright-field TEM micrographs (a, c) and HRTEM micrographs (b,d) of pure ZnO and Pd-loaded (1 mol%) ZnO NPs. The insets in (b) and (d) respectively show the SAED patterns of the pure and Pd-loaded (1 mol%) ZnO NPs. A closer analysis of the HRTEM images obtained from the pure ZnO sample [Fig. 4(b)] indicated the presence of lattice fringes with an inter-plannar spacing of 0.254 nm, which corresponded to the (101) planes of hexagonal ZnO. In contrast, the HRTEM image of the Pd-loaded ZnO sample [Fig. 4(d)] confirmed the co-existence of ZnO and Pd nanocrystals with inter-planar spacings of 0.248 nm and 0.225 nm between the lattice fringes corresponding to the (101) and (111) planes of hexagonal ZnO and face centred cubic Pd, respectively. In addition, the corresponding selected-area electron diffraction (SAED) patterns of the pure and Pd-loaded ZnO NPs shown in the insets in Fig. 4(b,d) confirmed that the nanograins are polycrystalline in nature, thereby confirming the previously discussed XRD results. Furthermore, the well-defined Laue spots indicate that the samples are crystalline in nature, while the observed diffraction rings confirm the polycrystalline nature of the ZnO NPs exhibiting a würtzite hexagonal structure. From the SAED patterns, average lattice constants of a = 0.332 ± 0.016 nm and c = 0,535 ± 0.027 nm were determined for the pure ZnO NPs, whereas the Pd-loaded ZnO (1 mol%) nanostructures showed no discernible change in a ( = 0.331 ± 0.017 nm), but a definite change in c was observed, with a calculated value of 0,522 ± 0.026 nm. this agrees well with values calculated from the XRD results.

**Figure 5:**
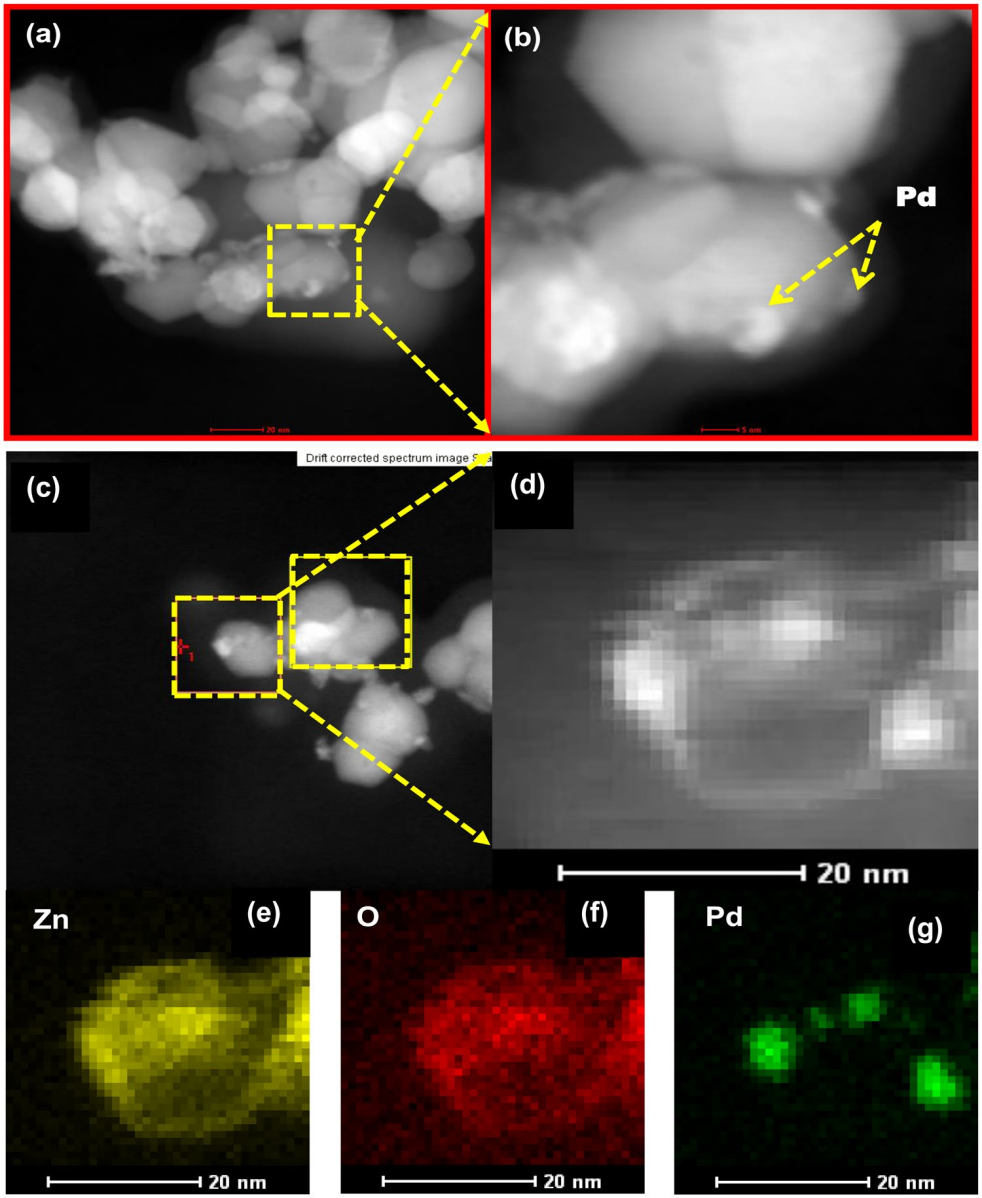
(**a**,**b**) HAADF-STEM micrographs, (**c**) drift corrected area indicated by the bigger square, and area for collection of the spectral image map indicated by the smaller square and arrows; (**d**) resultant spectral image collected from area identified in (**c**); (**e**–**g**) elemental maps of Pd-loaded (1 mol%) ZnO NPs.

To further confirm the presence and distribution of Pd on the ZnO surfaces, dark field HAADF images and STEM EDS SI were collected, as shown in Fig. 5(a–g). More specifically, Fig. 5(a,b) show the HAADF images of the Pd-loaded (1 mol%) ZnO nanostructures, where the contrast of the incoherent high-resolution HAADF images is directly proportional to the atomic number (Z) of the sample, with elements with greater Z exhibiting brighter contrast. Thus, the HAADF-STEM micrograph shown in Fig. 5(a) revealed the presence of clustered and aggregated nanograins of ZnO and Pd. A closer inspection of the corresponding magnified HAADF-STEM micrograph shown in Fig. 5(b) (selected area marked by a yellow line), showed the dispersion of small particles of Pd on the surface of the larger ZnO particles. To gain further insight on the distribution of Pd on ZnO, EDS elemental mapping was carried out and results are shown in Fig. 5(e–g). Figure 5(d) shows the resultant spectral image of the area identified in Fig. 5(c) and the corresponding elemental maps of Zn [Fig. 5(e)], O [Fig. 5(f)], and Pd [Fig. 5(g)]. These elemental maps revealed a uniform distribution of Zn, O, and Pd, thereby confirming that ZnO exists in a uniform phase and the relatively samller Pd nanocrystals are randomly distributed on the surface of the ZnO NPs.

**Figure 6:**
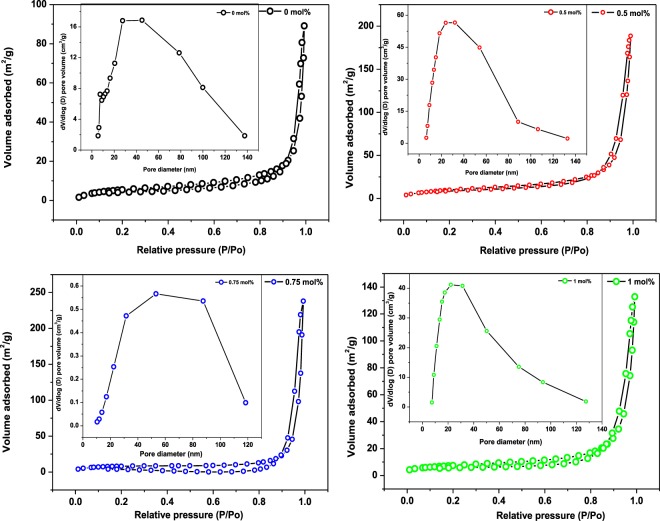
Nitrogen adsorption-desorption isotherms and the corresponding pore size distribution of the (**a**) 0, (**b**) 0.5, (**c**) 0.75 and (**d**) 1 mol% Pd-loaded ZnO NPs.

To gain more understanding about the porous nature of the produced pure and Pd-loaded ZnO products, N_2_ adsorption-desorption measurements were conducted. Figure 6 depicts the N_2_ adsorption-desorption isotherms along with the Barret-Joyner-Halenda (BJH) pore size distribution plots (see insets of Fig. 6) of the pure and Pd-loaded ZnO NPs. All the isotherms can be classified as the IV type isotherm according to IUPAC. Both the pure and Pd-loaded ZnO samples show a distinctive H3 type hysteresis loop at relatively high pressures for all isotherms, which is related to the filling and emptying of mesopores by capillary condensation. The specific surface area and pore size values for all Pd-loaded ZnO samples were found to be higher than that of pure ZnO samples as shown in Table 2, Such increase in both the surface area and pore size diameter values following addition of Pd on the ZnO NPs indictates that more active sites develops through doping by Pd whereas high porosity provides fast and efficient diffusion of target gas molecules thus playing a significant role in improving gas sensitivity during gas sensing.

**Table 2:** Summary of the BET surface area, pore volume and pore diameter of the pure and Pd-doped ZnO NPs.

Sample (mol%)	Surface area (m²/g)	Pore volume (cm³/g)	Average pore size (nm)
0	21.8053 ± 0.2048	0.11	20.64
0.5	39.7269 ± 0.2266	0.25	25.56
0.75	29.7687 ± 0.4920	0.29	39.69
1	26.9675 ± 0.0574	0.18	26.15

### Determination of chemical states by XPS

**Figure 7:**
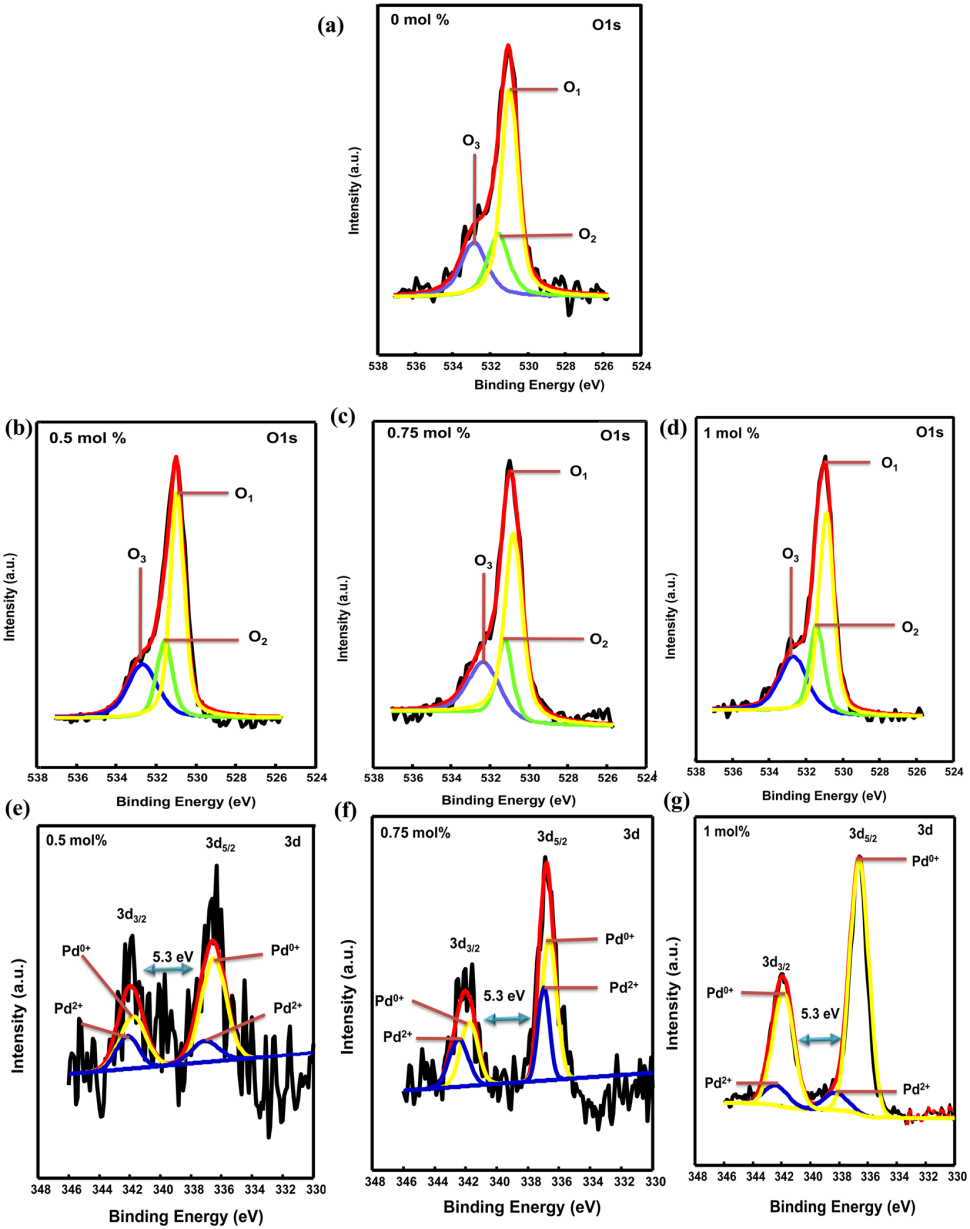
High-resolution XPS core level spectra of (**a**–**d**) O 1 s and (**e**–**g**) Pd 3d of the pure and Pd-loaded ZnO samples, respectively.

To further confirm the presence of Pd in the ZnO and probe the chemical states of the elements present in both pure and Pd-loaded ZnO NPs, XPS analyses were performed. Therefore, Fig. 7(a–d) shows the high-resolution XPS spectra for the O 1 s core level of the pure and various Pd-loaded ZnO NP, where the broad and asymmetric nature of the signals, indicates the presence of multiple oxygen species. To verify this assumption, each spectrum was partitioned into three peaks by multiple Gaussian fitting to give peaks at 530.9, 531.5, and 532.8 eV, denoted as O_1_, O_2_, and O_3_, respectively. More specifically, the peak at 530.9 eV was attributed to the O^2−^ ions of the lattice oxygen in ZnO^26,30^, while those at 531.5 and 532.8 eV were attributed to the O^2−^ ions of the surface hydroxyl groups and specific chemisorbed oxygen species on the ZnO surface (e.g., O^−^, O^2−^ or ${{\rm{O}}}_{2}^{-}$) respectively^26,30^. Indeed, the changes in the intensity of the O_2_ peak may be in connection with the changes in the content of oxygen defects such as oxygen vacancies (V_O_). Besides, the increase in the content of V_O_ is known to improve the formation of oxygen species since they can act as active sites for the chemisorption and ionization of oxygen on the surface of ZnO during gas sensing, thus contributing to the enhancement of sensors performance. On the other hand, the adsorbed oxygen functionalised as ionsorbed oxygen (i.e., O^−^, O^2−^ or ${{O}}_{2}^{-}$) has been demonstrated to play an important role in reducing conductivity by capturing the conducting electrons from ZnO. As the adsorbed oxygen species are regarded as imperative to the gas sensing mechanism, the content of this component is therefore of particular importance to interpret any differences in the sensing performance. In the present study the relative intensities of both O_2_ and O_3_ peaks were found to increase with increasing Pd content as shown in Table 3.

**Table 3:** Pd^2+^/Pd^0^ ratios and oxygen vacancies of Pd-loaded ZnO NPs from XPS spectra.

mol%	O_1_	O_2_	O_3_	O_2_/O_1_	Pd^2+^	Pd^0^	Pd^2+^/Pd^0^
	Area (%)	Area (%)	Area (%)		Area (%)	Area (%)	
0	57.3	19.8	22.9	0.35	—	—	—
0.5	54.4	20.4	25.2	0.37	25.05	74.96	0.33
0.75	50.3	22.9	26.8	0.46	37.46	62.53	0.56
1	50.6	22.5	26.9	0.44	10.88	89.12	0.12

In addition, the high-resolution spectra for the Pd 3d core level was recorded and is shown in Fig. 7(e–g), where detailed information regarding the chemical state of the loaded Pd can be found. In this case, the Pd 3d spectra of all Pd-loaded ZnO samples displayed doublet at 335.9 and 341.2 eV for Pd 3d_5/2_ and Pd 3d_3/2_, respectively, which correspond to Pd metal and exhibit spin-orbit doublet with separation values of 5.3 eV^30–32^ It is also apparent from this figure that both contributions are asymmetric to some extent, thereby demonstrating the presence of more than one Pd species in the near surface region of the ZnO NPs^24^. To confirm this assumption the two doublet (i.e., Pd 3d_5/2_ and Pd 3d_3/2_) were fitted by multiple Gaussians functions, and the fitted peaks of Pd 3d_5/2_ positioned at 335.9 and 336.7 eV were assigned to surface Pd° (Pd metal) and Pd^2+^ (PdO), respectively^24,26,32,33^. The presence of these two Pd species strongly demonstrates that a portion of the Pd is loaded on the ZnO surface, while the remainder is doped into the ZnO lattice. Indeed, this corresponds with the previously discussed XRD and TEM results.

The relative percentage concentration of Pd° and Pd^2+^ valence state for both Pd-loaded ZnO NPs were also calculated using Eqs (1) and (2).

1 $$[P{d}^{0}]surf=[P{d}^{0}]/[[P{d}^{2+}]+[P{d}^{0}]]$$

2 $$[P{d}^{2+}]surf=[P{d}^{2+}]/[[P{d}^{2+}]+[P{d}^{0}]]$$

These relative percentage concentration of both the Pd° and Pd^2+^ valence states were calculated using amounts of the integrated peak intensity areas corresponding to Pd° and Pd^2+^ oxidation spectra, respectively. In this case, [*Pd*^0^] *surf* and [*Pd*^2+^] *surf* represents the amount of Pd° and Pd^2+^ in all Pd-loaded ZnO NPs and the relative percentage amounts of each of these species are presented in Table 3. The relative contents of Pd^2+^ state for 0.5, 0.75, and 1 mol% Pd-loaded ZnO NPs were found to be 25, 38, and 11%, respectively and this strongly suggests that the surface of the 0.75 mol% Pd-loaded sample has been highly reduced. As a result, the amount of Pd^2+^ state is larger in 0.75 mol% Pd-loaded sample and this triggered the creation of more oxygen vacancy defects in ZnO lattice which is further confirmed through deconvolution of O 1 s XPS spectra as shown in Table 3 where the 0.75 mol% Pd-loaded sample displayed higher relative concentration of oxygen vacancies defects.

### NH_3_ gas sensing performance

As the operating temperature of a gas sensor has a significant influence on gas response, its effect on the sensing performance of both pure and Pd-loaded ZnO NPs-based sensors was investigated to determine the optimal operating temperature. The responses of the pure and Pd-loaded ZnO NPs-based sensors exposed to 40 ppm NH_3_ at a range of operating temperatures from 250 to 400 °C were recorded and are presented in Fig. S2 (see supplementary information). From the obtained response curves, it is evident that the sensing signals of all the sensors initially increased upon increasing the working temperature, prior to reaching a maximum at 350 °C, and all Pd-loaded ZnO sensors exhibited superior responses to that of the pure ZnO sensor over all operating temperatures examined. However, a significant drop in sensor response was observed upon further increasing the operating temperature to 400 °C. Such variation in the sensor responses with operating temperature can be associated with the adsorption–desorption kinetics of the analtye gas on the pure ZnO and Pd-loaded ZnO NPs-based sensor surfaces^34^. In actual facts, it has been previously reported that the response of an SMO-based sensor towards a certain target gas depends on the rate of the chemical reaction taking place on the sensor surface, in addition to the rate of gas molecule diffusion to the surface^35^. At low working temperatures, the response signal is restricted by the rate of the chemical reaction, while at high working temperatures, it is restricted by the rate of target gas molecules diffusion. In this context, the poor sensor response observed herein at low operating temperatures can be attributed to the lower thermal energy of the NH_3_ gas molecules in the reaction with surface-adsorbed oxygen species. In addition, the increase in sensor response signals upon increasing the operating temperature is likely due to an increase in the number of surface electrons through thermal activation, which in turn results in the increased adsorption of oxygen species and NH_3_ molecules on the active sites of ZnO. Furthermore, the sharp reduction in sensor response upon further increasing the operating temperature is likely due to the inaccessibility of active sites for NH_3_ adsorption. Furthermore, at operating temperatures higher than 350 °C, increased oxygen dissociation is observed, thereby permitting its adsorption on the active sites of ZnO, and reducing the number of active sites available for the adsorption NH_3_ molecules^8^.

**Figure 8:**
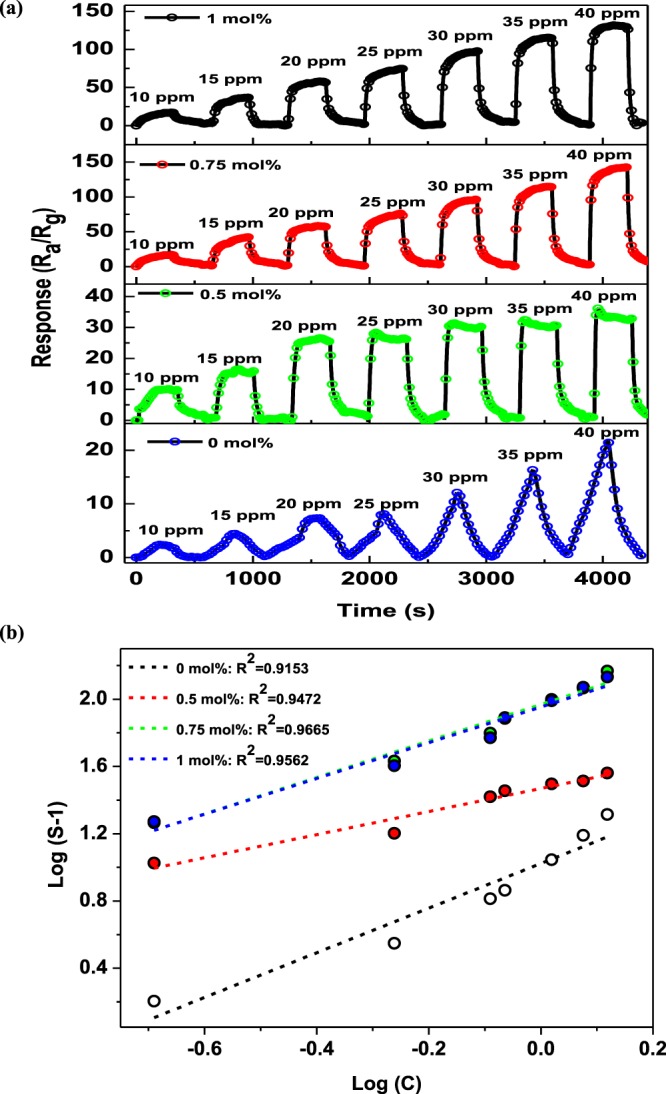
(**a**) Transient response curves, and (**b**) corresponding log (S-1) *vs* log (C) plots of the pure and Pd-loaded ZnO NPs based sensors in the presence of various NH_3_ concentrations at 350 °C.

To understand the sensing properties of the various sensors towards a range of NH_3_ concentrations, transient response measurements were performed. Thus, Fig. 8(a) shows the dynamic response/recovery transient curves of the pure and Pd-loaded ZnO NPs-based sensors obtained in the presence of 10−40 ppm NH_3_ at 350 °C. As expected, both sensors exhibited a gradual increase in response upon increasing the NH_3_ concentration, with a sharp drop in response back to the baseline being observed after the removal of NH_3_ from the system. However, the response-recovery curves of the 0 mol% ZnO based sensor displayed a relatively delayed/slow response behaviour which is indicative of abundance of reaction sites for adsorbed oxygen species available to react with NH_3_ gas molecules at a specific concentration. The delayed/slow response trend observed for 0 mol% based sensor in this case is one of the main drawbacks signifying the very slow adsorption and desorption of NH_3_ gas molecules on the ZnO surface thus leading to high/recovery times. Addition of Pd metal nanoparticles has been demonstrated to fast track the adsorption and desorption of target gas molecules leading to rapid response/recovery times for the sensors owing to its catalytic activity. For this reason, as observed from Fig. 8(a), the responses of all Pd-loaded based sensors were found to increase and reached a saturation point at a certain value upon NH_3_ exposure, then rapidly decreased after the removal of NH_3_ from the chamber as the sensors recover back to baseline.

Allthough all sensors demonstrated good sensing capabilities towards NH_3_ over the concentration range of interest, Pd-doping significantly enhanced the NH_3_ response, with the sensor containing 0.75 mol% Pd-loaded ZnO NPs-based sensor exhibiting the highest response as compared to the rest of the sensors. It has been previously reported that the response of an SMO-based sensor, such as ZnO, can be empirically expressed as:

3 $${S}_{g}=1+a{C}_{g}^{b}{\rm{q}}$$

where C represents the concentration of NH_3_, while *a* and *b* are constants defining the type of gas sensor and sensing material employed^20,26,36^. In this case, the value *b* relies on the charged state of the chemisorbed oxygen species, and can be either 0.5 (*O*^2−^) or 1 (*O*^−^), depending on the surface interactions taking place between the NH_3_ molecules and the chemisorbed oxygen species^20,36^. Thus, when the value of *b* is known, the indentity of the oxygen species adsorbed on the ZnO NPs can be speculated, and Eq. 4 can be re-written as follows:

4 $$\mathrm{log}({S}_{g}-1)=\,\mathrm{log}\,a+b\,log\,{C}_{g}$$

Figure 8(b) further depicts the logarithms of the responses for the pure and Pd-loaded ZnO NPs-based sensors *vs* the logarithm of the NH_3_ concentration at the optimum operating temperature of 350 °C. As indicated the responses of all sensors exhibited a linear relationship with the NH_3_ concentrations ranging from 10 to 40 ppm. This finding corresponds with previously reported results for similar systems^20,26,36^. In addition, it should be noted that the continuous increase in response at increasing NH_3_ concentrations arises from a low target NH_3_ concentration resulting in a lower surface coverage of NH_3_, which in turn reduces the number of surface reactions taking place between the gas molecules and the adsorbed oxygen species at the surface. Moreover, a high concentration of target gas molecules enhances the surface reaction owing to the increased surface coverage^16^.

**Figure 9:**
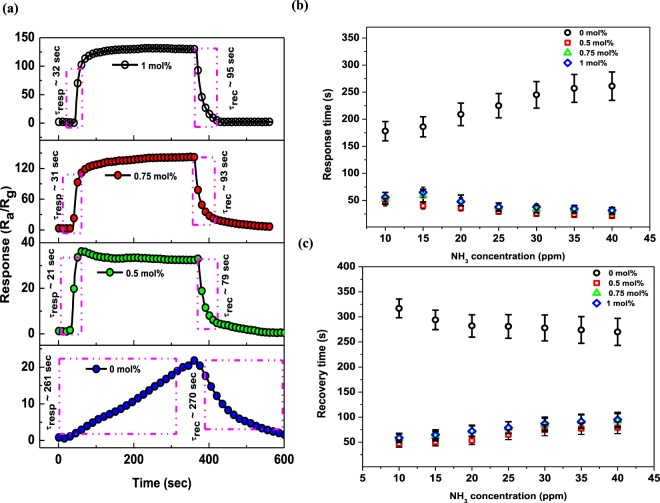
(**a**) Sensor response characteristics displaying the response/recovery times, and (**b**) the response and (**c**) recovery times of the pure Pd-loaded ZnO-based sensors to 40 ppm NH_3_ at an operating temperature of 350 °C.

The catalytic effect of Pd on the ZnO NPs has been found to not only contribute to an enhanced response and lower detection limit, but also to enhance the response kinetics (i.e., the response/recovery times). Indeed, previous studies have demonstrated that Pd loading onto SMOs can promote the adsorption and desorption of target gas molecules, thereby leading to rapid response/recovery times for the sensors^37^. In this context, Fig. 9(a) shows the dynamic response characteristics of the various sensors towards 40 ppm NH_3_ at 350 °C. As indicated, both the response and recovery times reduced significantly upon the introduction of Pd into ZnO. In the context of the response time, the sensing reaction to a reducing gas, such as NH_3_, is known to involve the diffusion of target gas molecules onto the ZnO surface and their subsequent oxidation by adsorbed oxygen species (*i*.*e*.,*O*^−^ or *O*^2−^). As such, the shortening of response times upon increasing the NH_3_ concentration [see Fig. 9(b)] is likely due to the increasing concentration gradient of the analyte gas. In adition, the incorporation of Pd can result in an abundance of ionsorbed oxygen species, which is advantageous in the oxidation of NH_3_^38^. In contrast, recovery times for the pure ZnO sensors decrease upon increasing the NH_3_ concentration and this is likely due to a delay in the recovery reactions caused by large numbers of vacant states. However, Pd-loaded ZnO tends to contain a large quantities of vacant states compared to the pure ZnO, and yet this material exhibited shorter recovery times in the current study [see Fig. 9(c)]. We except that this anomaly is due to the catalytic activity of Pd promoting the rapid dissociation of oxygen molecules over the Pd particles, which then results in the fast diffusion and adsorption of ions-orbed oxygen onto the ZnO surface. Indeed, rapid response/recovery times have also been reported by Chang *et al*.^31^ for Pd-decorated ZnO nanorods, while Trung *et al*.^39^ attributed the ultrafast response/recovery times of Pd-doped SnO_2_ nanowires to the catalytic activity of Pd nanoparticles, which led to acceleration of the reactions between the analyte gas molecules (CO) and the pre-adsorbed oxygen species ($i.\,e.\,,{O}_{2}^{-}$, *O*^−^, *O*^2−^).

### NH_3_-sensing mechanism of the pure and Pd-loaded ZnO NPs

The gas sensing capability of the ZnO sensor is governed by a change in the sensor electrical resistance, which is in turn caused by the adsorption/desorption of gas molecules at the ZnO surface. Generally, upon the exposure of pure ZnO to air, oxygen molecules are initially adsorbed on the surface of the active layer where they act as electron acceptors. These adsorbed oxygen species become ionised to chemisorbed oxygen species ($i.e.,\,{O}_{2}^{-}$, *O*^−^, and *O*^2−^) by capturing electrons from the conduction band of ZnO, thus resulting in an increase in electrical resistance. It is important to note that the chemisorbed oxygen species are strongly dependent on the operating temperature. For example, at operating temperatures below 130 °C, chemisorption of the stabilised ${{O}}_{2}^{-}$ takes place, while at operating temperatures between 130 and 300 °C, *O*^−^ is stabilised and chemisorbed onto the sensing material surface, and at operating temperatures higher than 300 °C, the stable *O*^2−^ is chemisorbed^36,40^. In the system reported herein, an operating temperature of 350 °C was employed for the detection of NH_3_, and so we could assume that *O*^2−^ was chemisorbed onto pure ZnO NPs- based sensor surface according to the following reaction:

5 $${O}_{2(ads)}^{-}+e\to 2{O}_{(ads)}^{2}$$

**Figure 10:**
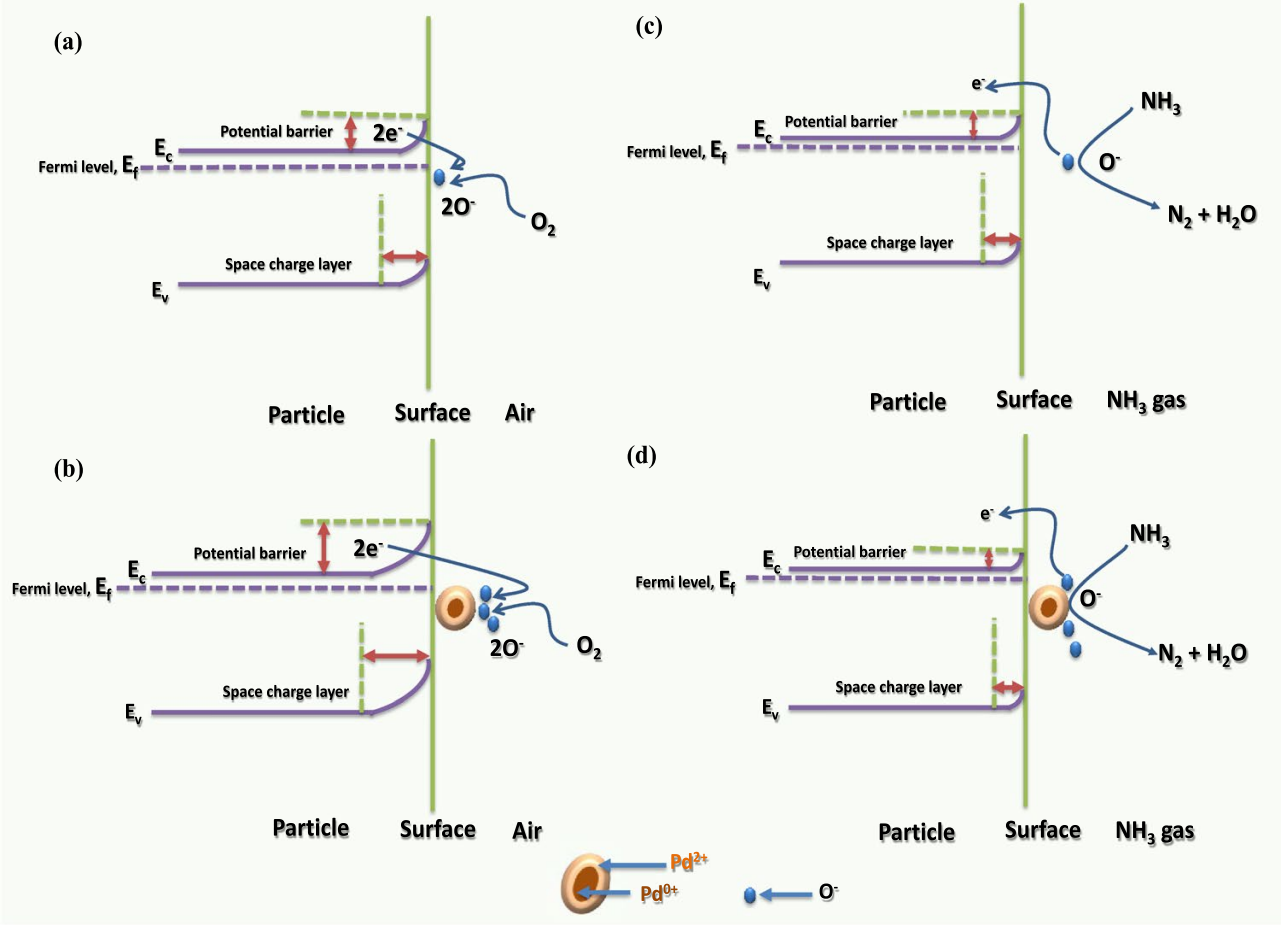
Schematic band diagrams of the pure ZnO NPs upon exposure to (**a**) air and (**b**) NH_3_, and corresponding band diagrams for the Pd-loaded ZnO NPs-based sensors upon exposure to (**c**) air and (**d**) NH_3_.

Therefore, when n-type SMOs such as ZnO are exposed to air, a region depleted of charge carriers (known as the depletion region) at the ZnO sensor interface is generated due to differences in the original Fermi levels of the materials (see Fig. 10(a))^21,41^. However, upon the exposure of pure ZnO to a reducing gas such as NH_3_, the adsorbed gas molecules come into contact with the chemisorbed oxygen species (*O*^−^) to form H_2_ and H_2_O, as per the following reaction:

6 $$4N{H}_{3(ads)}+3{O}_{2(ads)}^{-}\to {N}_{2(ads)}+6{H}_{2}{O}_{(ads)}+3{e}^{-}$$

As a result, the electrons trapped by the surface oxygen species are released back into the conduction band of ZnO, thereby reducing the electrical resistance [see Fig. 10(b)]^37^.

Upon exposure of Pd-loaded ZnO sensor to air, the oxygen molecules adsorbed on the Pd surface trap free electrons from the conduction band of ZnO, thereby creating a Pd^0^/Pd^2+^ redox couple with an electronic potential 5.5 eV below the vacuum level^31^. This leads to the generation of a depletion layer close to the Pd/ZnO interface, which originates from the large work function of the Pd^0^/Pd^2+^ redox thus reducing the conducting channel [see Fig. 10(c)]. In addition, upon exposure of a Pd-loaded ZnO sensor to NH_3_ gas, the adsorbed oxygen molecules initially react with the NH_3_ molecules to release trapped free electrons back to Pd, which results in the reduction of Pd^2+^ to Pd^0^, thereby reducing the work function of the Pd ions to 5.1 eV^31^. This results in an inward shift of the Fermi level of ZnO, which in turn leads to a reduction in the depletion layer at the Pd/ZnO interface [see Fig. 10(d)] causing a decrease in electrical resistance. These transformations thereby account for the observed improvement in sensor response following Pd loading.

**Figure 11:**
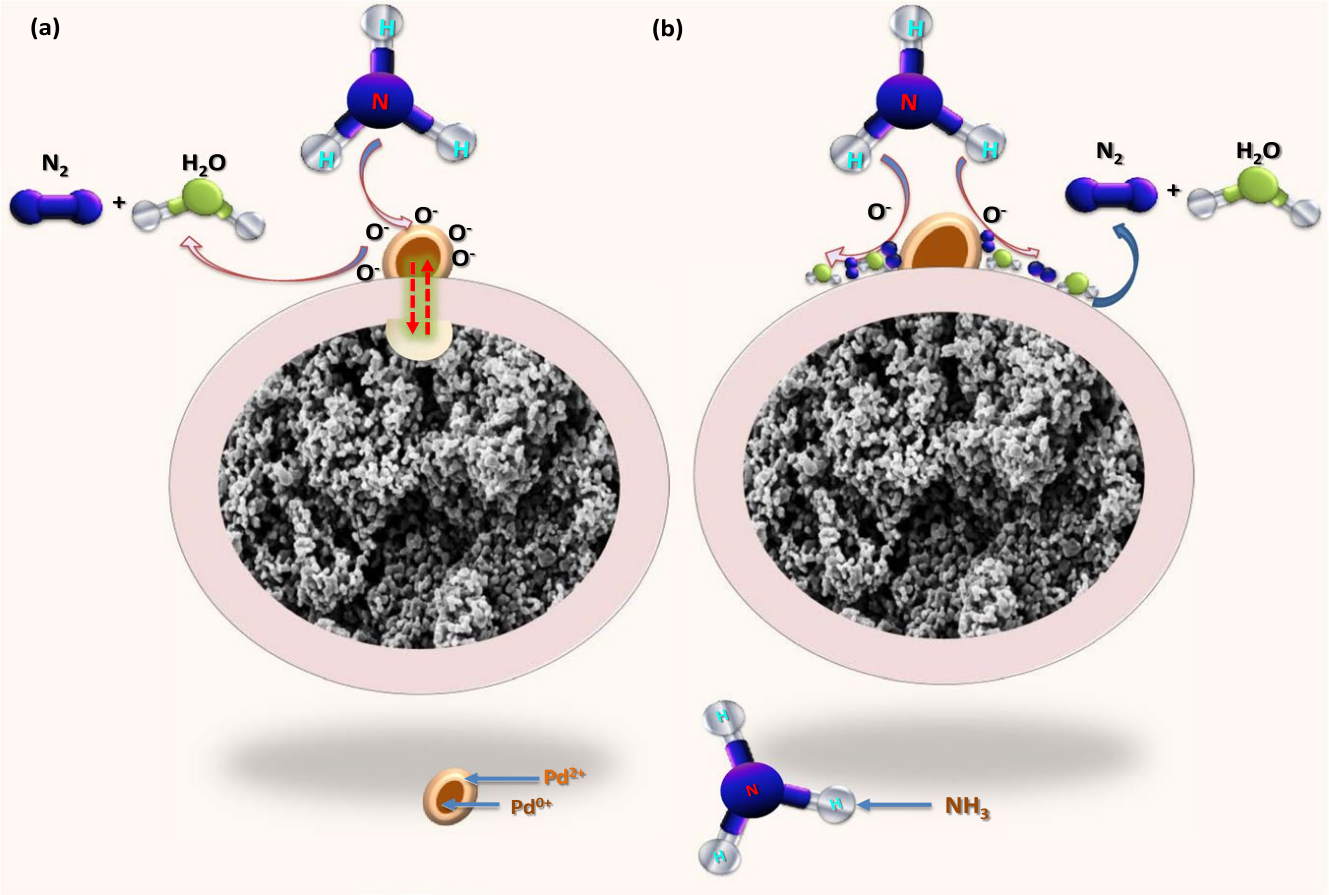
Schematic representations of (**a**) the electronic sensitisation and (**b**) the chemical sensitisation methods for the Pd-loaded ZnO sensors.

In this context, two types of mechanisms have been considered to explain the observed enhancement in sensors response following Pd loading into the ZnO NPs, namely electronic sensitisation and chemical sensitisation^42,43^. Based on the XPS results, it was assumed that Pd was oxidised to give PdO following annealing at 500 °C, as the presence of both Pd^0+^ and Pd^2+^ phases was observed. Indeed, similar findings were reported by Zeng *et al*.^42^ and Jiao *et al*.^43^ for Pd sensitised flower and rod-like ZnO nanostructures, respectively. Such variations in the chemical or oxidation states of Pd nanocrystals result in the formation of a depletion layer close to the Pd/ZnO interface. As previously discussed, the presence of an air atmosphere to the formation of a PdO phase, which acts as a strong acceptor of electrons originating from SMOs^44^. This creates a depletion layer at the interface between the ZnO NPs and the Pd nanocrystals, which results in electron transfer between ZnO and Pd/PdO as shown in Fig. 11(a). This is known as electron sensitization^44,45^.

In the case where oxidised Pd (PdO) comes into contact with NH_3_ molecules, it is then reduced back to Pd according to the following reaction:

7 $$3PdO+2N{H}_{3}\to 3Pd+3{H}_{2}O+{N}_{2}$$

Acting as a catalyst, PdO then promptes the dissociation of NH_3_ molecules into *H*_2_*O* and *N*_2_ active radicals through the spill-over effect, which is explained in detail elsewhere^15,16,42,46^. During this process, the NH_3_ molecules initially react with PdO prior to spilling-over to the ZnO surface to react with the adsorbed oxygen species. As a result, additional oxygen species are being created to react with NH_3_ gas molecules (see Fig. 11(b)) hence the improved NH_3_ sensing performance following Pd loading.

Finaly, based on the surface reaction related gas sensing phenomenon, the response values of the ZnO NPs based sensors can also be influenced by the specific surface areas. Generally, higher surface area offer additional active sites for adsorption of gas molecules and this often lead to improved sensor response values^7^. The BET analysis conducted via nitrogen adsorption revealed larger specific surface area values for all Pd-loaded ZnO NPs as compared to that of the pure ZnO NPs. However, it was noticed that the 0.5 mol% loading level showed the highest surface area while its pore diameter was found to be the lowest as compared the rest of loading levels (see Table 2). This finding indicate that a higher surface area on its own does not lead to improved sensing but also high porosity or porous surface as it provide fast diffusion and mass transportation of adsorbed target gas molecules leading to sensor responses enhancement. This elucidates the observed improved sensing performance revealed by the 0.75 mol% Pd-loaded ZnO sensor in the current study.

Besides, larger surface area associated with smaller grain size has been demonstrated to result in the enhancement of the density of intrinsic defects which plays a major role in the sensing performance improvement of ZnO^7,19^. This arises from the fact that the presence of abundant defects more especially donor defects offer more channels for target gas molecules transportation resulting in sensor response increment. In the current study, analysis from XPS revealed the increase in the density of defects such as V_O_ with Pd addition. To verify this observation, correlation sensor responses of pure and Pd-loaded ZnO NPs with the relative concentration of O_2_/O_1_ from XPS was done. An increase in the sensing response with the relative concentration of defects was observed and is shown in Fig. S3 (see supplementary information). This behaviour denote the dependence of sensor responses on the intrinsic defects with 0.75 mol % Pd loading showing higher response and relative concentration of defects. Furthermore, transition from Pd^2+^ to Pd^0^ oxidation state also plays an important role as it can result in abundance of ionsorbed oxygen species and this is adventigeous the oxidation of NH_3_ thus resulting in enhanced sensor response. Based on this finding, synergetic effects of higher surface area as a result of smaller crystallite/particle size achieved with Pd loading (as confirmed by XRD and TEM analysis) which led to large number of intrinsic defects may not be excluded as a possible cause for enhanced sensor response with Pd addition in this study. Thus, the improved response characteristics in this case therefore indicate the promising future application of Pd-loaded ZnO NPs for NH_3_ detection.

**Figure 12:**
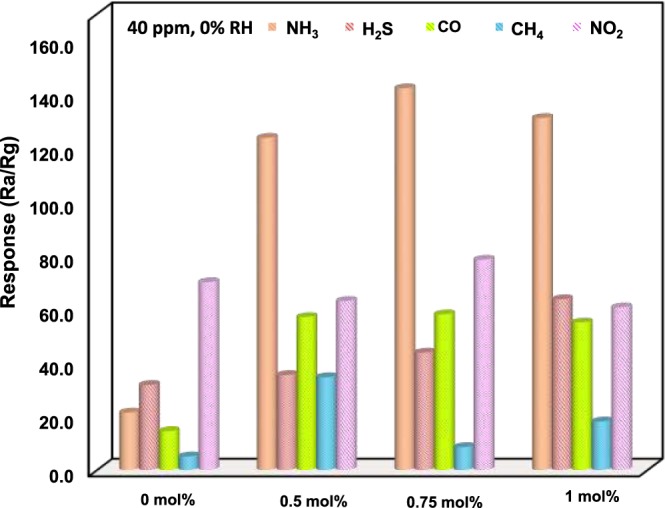
Comparison in responses of 0, 0.5, 0.75, and 1 mol% Pd-loaded ZnO based sensors to various gases with a same concentration at an optimal working temperature of 350 °C.

On the other hand, considering the fact that selectivity of a sensor towards a specific gas in the presence of other gases is a prerequisite for successful commercial/practical use, so both the pure and Pd-loaded ZnO based sensors were subjected to 40 ppm of different gases, namely, NH_3_, H_2_S, CO, CH_4_, and NO_2_ at the same operating temperatures of 350 °C. The results are depicted in the histogram of Fig. 12, respectively. Experimental findings obviously revealed that all Pd-loaded ZnO sensors displayed highest responses to NH_3_ with the 0.75 mol% Pd-loaded ZnO based sensor showing the highest response to NH_3_ as compared to the others. This means that Pd addition to ZnO improved the selectivity to NH_3_ suggesting that Pd-loaded ZnO NPs could be a promising gas active/sensing layer for detection of NH_3_ in real environments.

**Figure 13:**
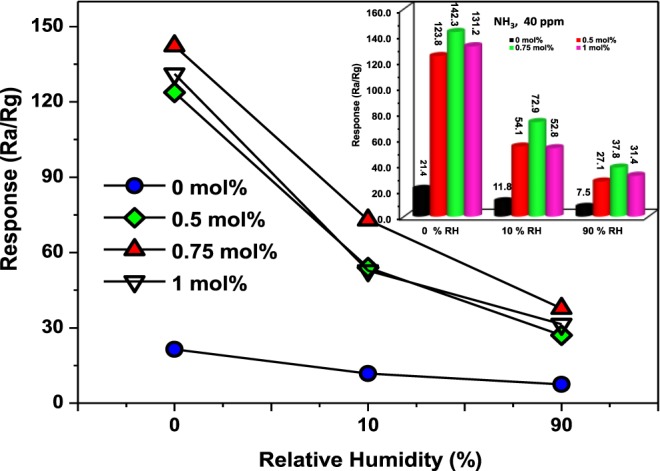
Responses of the pure and Pd-loaded ZnO NPs-based sensors to 40 ppm NH_3_ as a function of RH levels of 0, 10 and 90% RH. The inset presents the responses of the pure and Pd-loaded ZnO NPs-based sensors towards 40 ppm NH_3_ in dry air and under RH levels of 10 and 90% at 350 °C.

Apart from evaluation of interferering gases, the presence of water vapour molecules in the atmosphere is inevirtable and hence regarded as one of the crucial influencing factors that must be taken into consideration for practical use. Therefore, the comparison of response curves obtain350 °Cd towards 40 ppm NH_3_ both in dry and under humid atmospheric conditions of 10 and 90% at 350 °C for both the pure and Pd-loaded ZnO NPs-based sensors were recorded in the current study, as shown in Fig. 13. Indeed, a noticeable decrease in the response values for both the pure and Pd-loaded ZnO NPs-based sensors was observed upon 10 and 90% relative humidity (RH) levels^47^. In comparison to the obvious drop in sensor responses under relative humidities in this work, Li *et al*.^48^ reported a decrease in response values by 30% and 9% for the pure and Cr-doped WO_3_ nanofibres, respectively upon increasing the RH from 30 to 90%. Maswangwane *et al*.^49^ also found that both the undoped and In-doped ZnO displayed optimal response values at a RH level of 50% since further increase in RH levels up to 100% led to significant drop in response values of these sensors. The observed drop in sensor performance with increasing humidity levels in this work therefore arises from competition between the water vapor molecules and the NH_3_ molecules for the surface reaction sites, thereby limiting oxygen adsorption^13^. As a result, the number of oxygen species decreases at higher RH values, causing a disturbance in the sensor response signal towards NH_3_ gas.

**Figure 14:**
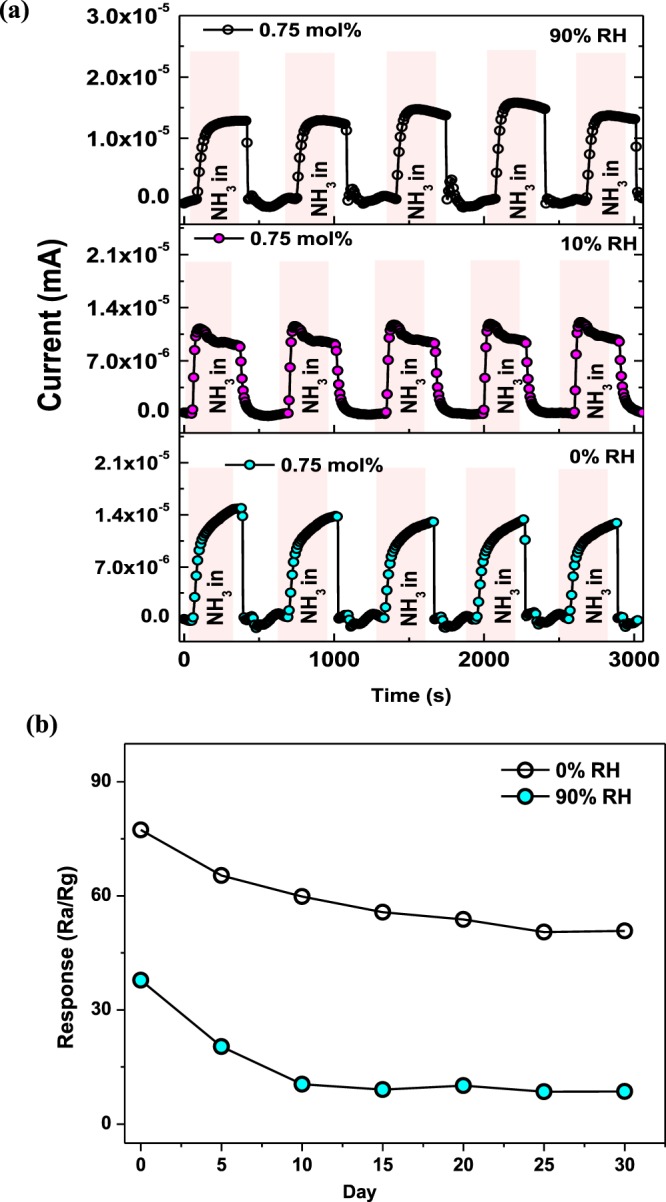
(**a**) Response-recovery cycles in dry air and under RH levels of 10 and 90% while (**b**) presents the long-term stability in dry air and under RH level of 90% of the 0.75 mol% Pd-loaded ZnO NPs-based sensors towards 40 ppm NH_3_ at 350 °C.

To further gain more insight about the potential applicability for the detection of NH_3_ gas, the reproducibility studies were conducted on a highly responsive Pd-loaded ZnO based sensor. For this reason, reproducibility of the 0.75 mol% Pd-loaded ZnO based sensor was investigated by repeatedly subjecting it to 40 ppm of NH_3_ gas at an optimal operating temperature of 350 °C in both dry and humid atmospheres of 10 and 90%. These tests were conducted five times under similar conditions to assess its repeatability charecteristics and the transient response-recovery curves showing five continuous reversible cycles are demonstrated in Fig. 14(a). It can be seen from this short-term stability study that the response-recovery curves acquired for five cycles are closely alike with a very slight fluctuation of electrical current suggesting a good reproducibility of the 0.75 mol% Pd-loaded ZnO based sensor. The long-term stability was also verified every 5 days for 30 days. It was realized that after 10 months of initial sensing tests, the sensor still maintained almost 65% of the original value which is an indication of good long-term stability. Even though the sensor displayed a decrease in the response signal at the first cycle as shown in Fig. 14(b). However, the five cycles showed good repeatability or stability behaviour further indicating good long-term stability fo this sensor.

## Conclusions

We herein described the prepartion of Pd-loaded ZnO NPs-based sensors via a chemical precipitation approach and examined the effect of Pd-loading quantities on the NH_3_ sensing capabilities in both dry and humid environments. Characterisation of the obtained ZnO NPs, both before and after Pd loading with 0.5, 0.75, and 1 mol% Pd was carried out, with X-ray diffraction revealing that the ZnO nanostructires are composed of a single phase würtzite structure, while high-resolution transmission electron microscopy confimed the random distribution of small Pd nanocrystals on the surface of ZnO nanoparticles. Following characterization, gas sensing studies were carried out, and all the Pd-loaded ZnO NPs-based sensors displayed improved response to NH_3_ in addition to rapid response-recovery times under both dry and humid conditions compared to pure ZnO NPs-based sensor at an optimal operating temperature of 350 °C. Further, the 0.75 mol% Pd-loaded ZnO NPs-based sensor displayed enhanced NH_3_ sensing performance in comparison to the rest of the sensors and this was explained in consideration of the high surface area, porosity and large number of intrinsic defects achieved with this Pd loading oncentration in addition to the catalytic activity of the Pd nanocrystals which served as direct adsorption sites for oxygen and acetone; thus catalytically activating the gas molecules spilling them over to the surface of the ZnO NPs. However a decline in the sensor performance under humid condition was also observed, due to competition between the water vapour and NH_3_ molecules for the surface active sites bearing adsorbed oxygen species. Based on these results, it is expected that the improved NH_3_ detection performance displayed by the Pd-loaded ZnO NPs-based sensors could lead to their application in for environmental monitoring and control, thereby contributing to a safer and healthier living conditions due to the control of environmental NH_3_ concentrations.

## Supplementary information

Supplementary information
