# Supplementary material for: A highly responsive NH3 sensor based on Pd-loaded ZnO nanoparticles prepared via a chemical precipitation approach
Source: Sci Rep. 2019 Jul 8;9:9881. doi: 10.1038/s41598-019-46247-z (PMC6614408; doi:10.1038/s41598-019-46247-z)
Supplement: Supplementary file 1 — Supplementary information [file 41598_2019_46247_MOESM1_ESM.docx]

**A highly responsive NH_3_ sensor based on Pd-loaded ZnO nanoparticles prepared via a chemical precipitation approach**

[G. H. Mhlongo](http://www.sciencedirect.com/science/article/pii/S0925400517312972#%21)^a,b*^, D. E. Motaung^a,b^, F. R. Cummings^c^, H. C. Swart^b^, [S.](http://www.sciencedirect.com/science/article/pii/S0925400517312972#%21) S. Ray^a,d^

*^a^ DST-CSIR National Centre for Nanostructured Materials, Council for Scientific and Industrial Research*

*Pretoria 0001, South Africa*

*^b^Department of Physics, University of the Free State, Bloemfontein ZA9300, South Africa*

*^c^Electron Microscopy Unit, University of the Western Cape, Bellville,*

*7535, South Africa*

*^d^Department of Applied Chemistry, University of Johannesburg, Doornfontein 2028, Johanneburg, South Africa*

**Supplementary Information**

**1.1 Determination of chemical states by XPS**

The high-resolution XPS spectra of the pure and various Pd-loaded ZnO NPs corresponding to the Zn 2p core level are presented in Fig. S1 show the spin-orbit splitting of the Zn 2p_3/2_ and Zn 2p_1/2_ core level states positioned at 1022.0 and 1045.1 eV, respectively, for both the pure and various Pd-loaded ZnO NPs. ^1^ The spin-orbit splitting of the observed Zn 2p doublet was 23.0 eV, which is consistent with the previously reported value.^2^

**Fig. S1.** High resolution XPS spectra of the pure and Pd-loaded ZnO samples corresponding to the Zn 2p core level.

**1.2 NH_3_ gas sensing performance**

Fig. S2 shows the responses of the pure and Pd-loaded ZnO NPs-based sensors exposed to 40 ppm NH_3_ at a range of operating temperatures from 250 to 400 °C.

**Fig. S2.** Effect of operating temperature on the response of pure and Pd-loaded ZnO sensors to 40 ppm of NH_3_.

**Fig. S3**. shows the correlation of sensor responses of the pure and Pd-loaded ZnO NPs with the relative concentration of Ob/Oa from XPS was done.

**Fig. S3.** Responses of pure and Pd-loaded ZnO NPs-based sensors versus XPS (Ob/Oa)

**References**

[1] Arunkumar, S., Hou, T., Kim, Y-B, Choi, B., Park, S. H., Jung, S., Lee, D-W. Au decorated ZnO hierarchical architectures: Facile synthesis, turnable morphology and enhanced CO detection at room temperature. *Sensor Actuat B-Chem* **243**, 990−1001 (2017).

[2] Chang, Y., Xu, J., Zhang, Y., Ma, S., Xin, L., Zhu, L., Xu, C. Optical properties and photocatalytic performance of Pd modified ZnO samples, *J* *Phys Chem C* **113**, 18761−18767 (2009).
